# 3′,4′-Dihydroxyflavonol Modulates the Cell Cycle in Cancer Cells: Implication as a Potential Combination Drug in Osteosarcoma

**DOI:** 10.3390/ph14070640

**Published:** 2021-07-03

**Authors:** José Miguel P. Ferreira de Oliveira, Joana Filipa D. Almeida, Maria Martins, Carina Proença, Helena Oliveira, Eduarda Fernandes, Conceição Santos

**Affiliations:** 1 LAQV, REQUIMTE, Laboratory of Applied Chemistry, Department of Chemical Sciences, Faculty of Pharmacy, University of Porto, 4050-313 Porto, Portugal; cproenca@ff.up.pt; 2 Department of Biology, Faculty of Sciences, University of Porto, 4169-007 Porto, Portugal; up201303752@edu.ff.up.pt (J.F.D.A.); up201306375@edu.fc.up.pt (M.M.); csantos@fc.up.pt (C.S.); 3 Department of Biology & CESAM, University of Aveiro, 3810-193 Aveiro, Portugal; holiveira@ua.pt; 4 LAQV, REQUIMTE, Faculty of Sciences, University of Porto, 4169-007 Porto, Portugal

**Keywords:** bone sarcoma, flavonoid, doxorubicin, anthracycline, drug dose reduction, cell cycle arrest, cyclins, cyclin-dependent kinases

## Abstract

New agents are demanded to increase the therapeutic options for osteosarcoma (OS). Although OS is the most common bone cancer in children and adolescents, it is considered a rare disorder. Therefore, finding adjuvant drugs has potential to advance therapy for this disease. In this study, 3′,4′-dihydroxyflavonol (DiOHF) was investigated to assess the effects in OS cellular models in combination with doxorubicin (Dox). MG-63 and U2OS human OS cells were exposed to DiOHF and Dox and tested for cell viability and growth. To elucidate the inhibitory effects of DiOHF, additional studies were conducted to assess apoptosis and cell cycle distribution, gene expression quantification of cell cycle regulators, and cytokinesis-block cytome assay to determine nuclear division rate. DiOHF decreased OS cell growth and viability in a concentration-dependent manner. Its combination with Dox enabled Dox dose reduction in both cell lines, with synergistic interactions in U2OS cells. Although no significant apoptotic effects were detected at low concentrations, cytostatic effects were demonstrated in both cell lines. Incubation with DiOHF altered cell cycle dynamics and resulted in differential cyclin and cyclin-dependent kinase expression. Overall, this study presents an antiproliferative action of DiOHF in OS combination therapy via modulation of the cell cycle and nuclear division.

## 1. Introduction

Osteosarcoma (OS) is a cancer type characterized by genetic heterogeneity and instability [1]. It is typically a high-grade malignancy with a rapid growth rate. The etiology of OS is multifaceted and poorly understood [2,3]. Conventional OS is the most frequent histologic type and accounts for about 75% of all cases. The classic form of OS is characterized by a high-grade mass of malignant mesenchymal cells with osteoid production and local tissue invasion. Conventional OS can be classified according to which matrix-producing cells dominate, resulting in osteoblastic, chondroblastic, or fibroblastic types [4]. In general, these conventional tumors behave similarly concerning the appearance and prognosis [4]. OS is a rare disease already classified as an orphan disease (orpha number ORPHA:668). It has an annual incidence rate of approximately 3.1 cases per million in the United States, accounting for less than 1% of all newly diagnosed cancers in adults and 3–5% of those in children. Although rare, it is the most common primary malignancy in adolescents outside leukemia and lymphoma, and the most common primary malignancy of bone in children [3,4,5,6]. Prior to the advent of chemotherapy, OS was generally a fatal disease although over the last 30 years, no significant improvements were achieved for metastatic OS [7]. Advances in chemotherapy and surgery allowed most patients to survive. However, a significant number of those affected with this type of cancer will most probably still develop fatal metastatic disease or serious complications of treatment, such as cardiomyopathy, related to drug resistance and toxicity [4,8]. These observations emphasize the need of further therapeutic advancements. Given the complexity of the disease, it is increasingly acknowledged that hitting a single target with a specific drug is not likely to be sufficiently disruptive for successful therapy.

The treatment modality that combines two or more therapeutic agents, known as combination therapy, is a cornerstone of cancer therapeutics. It is currently acknowledged that combination of anti-cancer drugs enhances efficacy when compared to monotherapy since it targets key pathways in a synergistic or additive manner. This approach is applied with the purpose to reduce drug resistance, while simultaneously providing anti-cancer properties, including the reduction of tumor growth and metastatic potential, the arrest of mitotically active cells, the reduction of stem cell populations, and also the induction of apoptosis [9]. The development of new combination/adjuvant drugs for rare diseases is typically a slow process, and this has raised awareness on drug repurposing or repositioning, i.e., investigation of new uses for drugs considered safe for humans. Drug repurposing decreases drug development time and cost, with cost reduction estimated in ca. US $2 billion per drug [10,11]. Databases such as the Repurposing Drugs in Oncology (ReDO) project have identified over 240 non-cancer prescribed drugs, of which 27% have one or more scientific publications showing activity in OS [12].

Concerning OS, bone health relies on the dynamic equilibrium between bone formation and degradation, and phenolic compounds show positive effects on the balance between osteoclastogenesis and osteoblastogenesis [13]. In previous studies, selected flavonoids were shown to induce osteoblastic differentiation and to inhibit osteoclastogenesis [14,15,16,17]. Flavonoids were also reported to induce several anti-OS activities, including cell cycle arrest, induction of apoptosis and inhibition of invasion and metastasis, suggesting a therapeutic potential [18,19,20,21,22,23]. Concerning current OS therapy, the anthracycline doxorubicin (Dox) is the most commonly prescribed agent to treat this cancer. However, the repeated administration of this anthracycline poses relevant cardiotoxicity risks originating from increased mitochondrial dysfunction and myocyte apoptosis [8]. In this respect, several flavonoids were reported to counteract Dox-induced toxicity, by decreasing oxidative stress and myocyte apoptosis [24,25]. 

3′,4′-Dihydroxyflavonol (DiOHF) belongs to the flavonoids class, a group of phenolic and heterocyclic compounds. Flavonoids are secondary metabolites naturally occurring in plants and largely found in foods and beverages, namely, fruits, vegetables, cereals, tea, coffee, and red wine [26]. DiOHF has been previously reported as possessing antioxidant properties, including the scavenging of superoxide and peroxyl radicals, and the inhibition of superoxide formation [27]. It was recently described that DiOHF increases apoptosis, and reduces the production of tumor necrosis factor (TNF)-α, as well as the DNA damage in rats with brain ischemia–reperfusion [28]. This synthetic flavonoid is also an investigational drug with reported vasorelaxant and chronotropic properties [29] and is currently investigated in Phase II clinical trials (registration ACTRN12618001692224) for the attenuation of paroxysmal atrial fibrillation in adults with an implanted cardiac device. Despite this, to the best of our knowledge, its role as inhibitor of cancer cell proliferation and as modulator of cell cycle progression is not reported. 

In the present study, DiOHF effects in OS cells were investigated in combination with the anthracycline Dox. Moreover, the main cytotoxic effects were investigated. For this, OS cell lines were exposed to DiOHF and analyzed for cytotoxicity, cell death, cell cycle dynamics and nuclear division rate. This study allowed the assessment of DiOHF, a drug with unidentified effects in cancer, as a potential drug for OS with synergistic interactions with Dox.

## 2. Results

### 2.1. DiOHF Inhibits Osteosarcoma Proliferation through Non-Apoptotic Mechanisms

**Figure 1:**
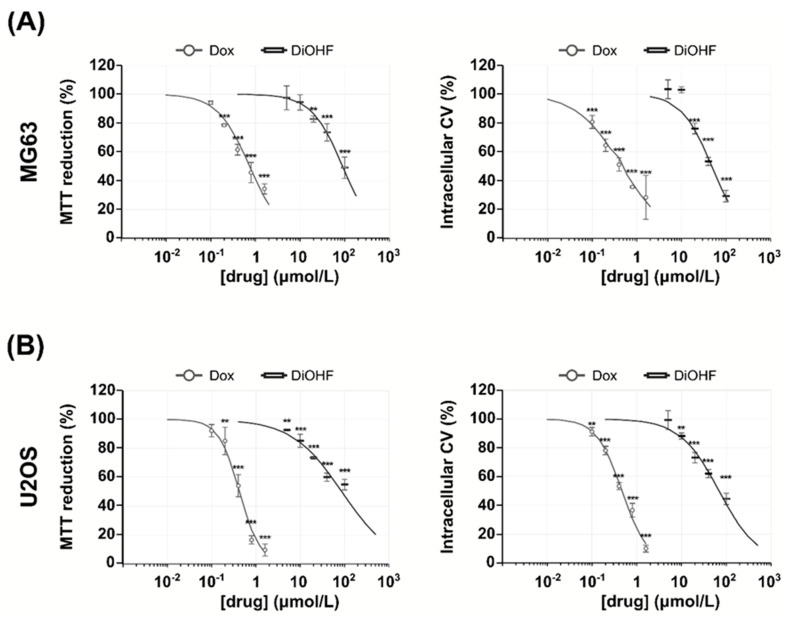
Cytotoxic effects of DiOHF. (**A**) MG-63 and (**B**) U2OS cells were incubated for 48 h with 5, 10, 20, 40 and 100 μmol/L DiOHF or 0.1, 0.2, 0.4, 0.8 and 1.6 μmol/L Dox. Cells were analyzed for cytotoxicity by MTT and CV assays. The data are mean ± SD (*n* = 3). Significant differences (asterisks) are shown relative to 0.1% DMSO control; ** *p* < 0.01, *** *p* < 0.001 (one-way ANOVA).

MG-63 and U2OS cell viability and growth determined by 3-(4,5-Dimethyl-2-thiazolyl)-2,5-diphenyl-2H-tetrazolium bromide (MTT) and crystal violet (CV) assays decreased with increasing DiOHF concentrations (Figure 1). In the MG-63 cell line (Figure 1A), after 48 h exposure, cell viability and growth (MTT and CV assays respectively) were significantly decreased at concentrations equal or above 20 μmol/L DiOHF. In the U2OS cell line (Figure 1B), MTT reduction was significantly decreased at all concentrations tested, while CV staining was decreased at DiOHF concentrations above 10 μmol/L. With respect to Dox, a significant decrease in MTT reduction was observed at Dox concentrations equal or above 0.2 μmol/L, while a decrease in CV staining was observed at all concentrations tested. From the dose-response curves shown in Figure 1, IC_50_ values were estimated (Table 1).

The subsequent assays of cell death, cell cycle dynamics and nuclear division were conducted at concentrations up to 40 μmol/L DiOHF, below the determined IC_50_ values. In order to examine the role of DiOHF in cell death by apoptosis and necrosis, a flow cytometry based annexin-V assay was performed. At concentrations up to 40 μmol/L DiOHF no significant effects were observed on the % of viable, apoptotic and necrotic cells (Figure S1).

### 2.2. DiOHF Enhances the Efficacy of The Anthracycline Doxorubicin (Dox) 

**Figure 2:**
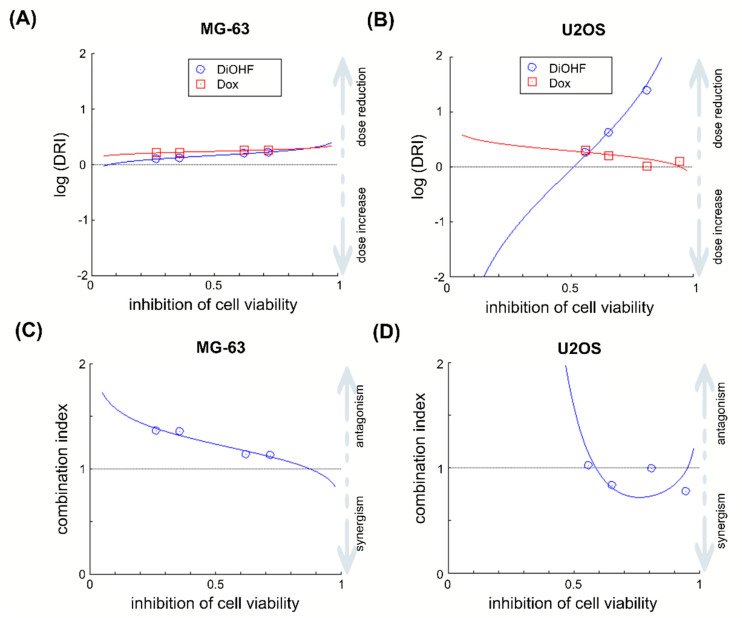
Combination effects of DiOHF and Dox (**A**,**C**) MG-63 and (**B**,**D**) U2OS cells were incubated for 48 h with DiOHF (39.5, 59.3, 133 and 200 μmol/L), Dox (0.222, 0.333, 0.75 and 1.125 μmol/L) or their respective increasing combinations. After MTT assay, a combination analysis was performed to identify for each combination (**A**,**B**) the dose reduction of each drug and (**C**,**D**) drug synergism.

Considering the IC_50_ values obtained in the MTT assay, a molar ratio of (200:1.125) was chosen for DiOHF–Dox mixtures. Based on MTT results (*n* = 3), the IC_50_ values for the MG-63 cell line were DiOHF: Dox = 98.5 (± 37.5): 0.55 (± 0.21) µM. For the U2OS cell line, IC_50_ values were DiOHF: Dox = 34.6 (± 3.6): 0.19 (± 0.02) µM. In both cell lines, dose reduction index (DRI) > 1 for all mixtures (Figure 2), which shows that in vitro DiOHF can reduce the Dox dose required to inhibit OS cell viability. Despite the Dox dose reduction achieved by DiOHF, the selected mixtures showed antagonistic effects on the inhibition of MTT reduction in the MG-63 cell line (Figure 2A). On the other hand, in the U2OS cell line, the same mixtures showed additive to synergistic effects in the inhibition of MTT reduction (Figure 2B).

### 2.3. DiOHF Induces Cell Cycle Arrest and Modulates Cyclin and Cyclin-Dependent Kinase Expression In Vitro

**Figure 3:**
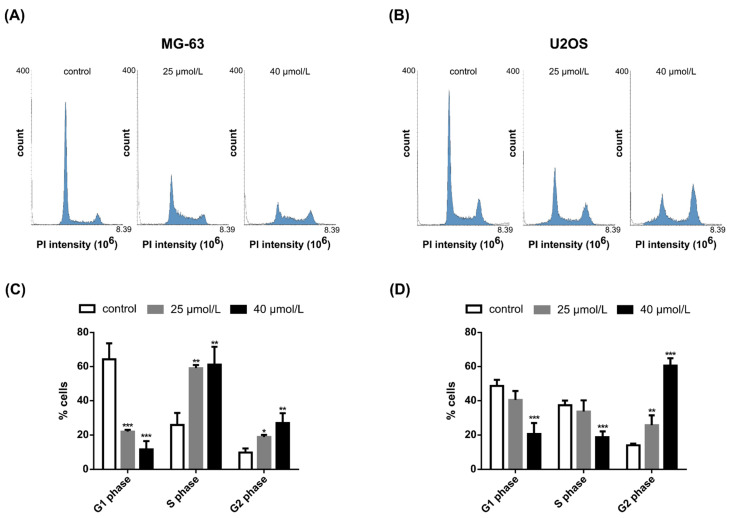
Effects of DiOHF on cell cycle distribution. MG-63 and U2OS cells were stained with propidium iodide and the cell cycle was analyzed by flow cytometry. Histograms representative of cell cycle distribution of (**A**) MG-63 and (**B**) U2OS cells. Cell cycle distribution (%) in (**C**) MG-63 and (**D**) U2OS cells. The data are mean ± SD (*n* = 3). Significant differences (asterisks) are shown relative to 0.1% DMSO control; * *p* < 0.05, ** *p* < 0.01, *** *p* < 0.001 (one-way ANOVA).

Compared to the control, after 48 h exposure, cell cycle distribution was significantly altered in both cell lines (Figure 3). Incubation with a low concentration of 25 μmol/L DiOHF showed more pronounced effects on the MG-63 cell line, when compared to the U2OS cell line. Upon exposure to DiOHF, the relative percentage of MG-63 cells in G1 phase decreased and the percentage of cells in S and G2 phase increased significantly. In U2OS cells, incubation with 40 μmol/L DiOHF decreased the cell population in G1 and S phases. Moreover, incubation with 25 μmol/L and 40 μmol/L DiOHF caused a significant increase in the percentage of cells in G2 phase (*p* < 0.01 and *p* < 0.001, respectively).

**Figure 4:**
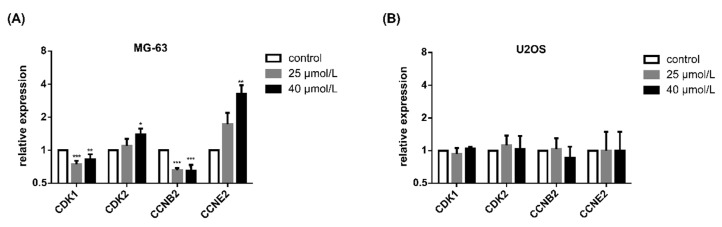
Effects of DiOHF on the gene expression of cell cycle regulators. Relative gene expression relative to control was calculated for (**A**) MG-63 and (**B**) U2OS cells exposed to DiOHF for 48 h. The data are mean ± SD (*n* = 3). Significant differences (asterisks) are shown relative to 0.1% DMSO control; * *p* < 0.05, ** *p* < 0.01, *** *p* < 0.001 (one-way ANOVA).

Under conditions identical to those investigated for cycle distribution, DiOHF altered the expression of selected cell cycle regulators (Figure 4). Considering the gene expression on each condition relative to DMSO control, in the MG-63 cell line incubation with DiOHF decreased the expression of cyclin-dependent kinase (Cdk)1- and cyclin B2-encoding genes and increased the expression of Cdk2 and cyclin E2-encoding genes, whereas in the U2OS cell line (Figure 4B) no significant changes were observed for these genes. 

### 2.4. DiOHF Inhibits Nuclear Division in Cytokinesis-Blocked Cells

**Figure 5:**
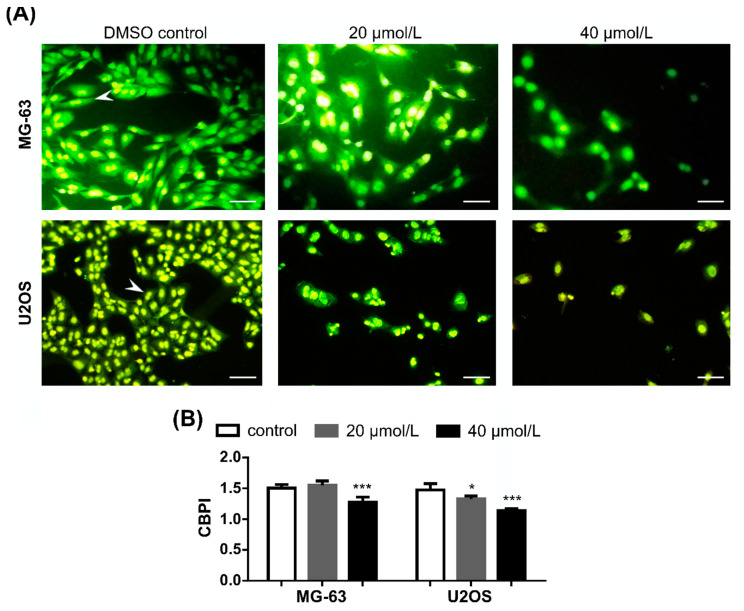
Nuclear division rate. Cells were exposed for 48 h, incubated with cytochalasin B for 40 h, fixed and stained with acridine orange. (**A**) fluorescence microscopy images of exposed cells. Arrow-heads: binucleate proliferating cells; scale bar: 100 μm. (**B**) cells were discriminated according to the number of nuclei (mononucleate, binucleate or multinucleate), and CBPI values were determined. The data are mean ± SD (*n* = 4). Significant differences (asterisks) are shown relative to 0.1% DMSO control; * *p* < 0.05, *** *p* < 0.001 (one-way ANOVA).

Following DiOHF exposure and after 40 h incubation with cytochalasin B, cell density was decreased in both cell lines (Figure 5A). Upon inhibition of cytokinesis, the experimental groups (exposed cells) showed higher frequency of mononucleate cells, indicating decreased nuclear division rates compared to the control. This cytostatic effect was significant for the highest concentration of DiOHF in the two cell lines, as indicated by decreased values of cytokinesis-block proliferation index (Figure 5B, *p* < 0.001).

## 3. Discussion

OS mainly affects people at young ages, and is the most common primary bone cancer. Although the chances of cure have significantly increased with surgery and cytotoxic chemotherapy, recurrent and refractory disease are still challenging, with limited therapeutic alternatives in current use and poor results [5]. As combination therapies might produce a stronger treatment for a disease compared to individual treatments, there are currently more than 10,000 clinical trials in progress, only in the United States, regarding the application of combination therapies for several diseases, namely cancer, infectious diseases, autoimmune, cardiovascular, metabolic, and neurological disorders. [30]. Despite this, drug combinations are still poorly explored in OS. In addition to the previously reported vascular [31,32] and antioxidant properties of DiOHF [32,33], as far as we know, this is the first study concerning the anticancer effects of this synthetic flavonoid, as well as its combination with Dox, for possible further application in the treatment of OS. In this work, DiOHF induced antiproliferative effects in MG-63 and U2OS cells, which were associated with cell cycle alterations and decreased nuclear division rates. Under the studied conditions, no relevant apoptotic or necrotic effects were detected at subtoxic DiOHF concentrations, an observation which supports inhibition of cell division at low concentrations. Moreover, in this study, DiOHF showed stronger inhibitory effects on cell density and protein amount (CV assay) compared to the metabolic MTT assay, pointing to stronger inhibition of cell division but not of metabolic reduction of MTT, e.g., by mitochondrial metabolism. Since DiOHF is a strong antioxidant, this raises the hypothesis that this compound enhanced the reductive metabolism in OS cells despite decreasing cell density. For this reason, it was important to investigate combinations with Dox in which reductive metabolism would not be enhanced by DiOHF.

Dox is the anthracycline most used in OS therapy. Although Dox is one of the most active drugs for the treatment of OS, some studies have shown that a subset of OS patients are resistant or may become unresponsive to this drug during chemotherapeutic treatment [34,35]. In addition, despite its main mechanism of action as a DNA intercalating agent and topoisomerase II inhibitor, its use is limited by severe side effects such as cardiotoxicity [8]. Thus, new therapeutic strategies, including the combination of Dox with other anti-OS molecules may overcome this problem [35]. In this study, to assess drug interactions with Dox, MTT assays were used to compare DiOHF with Dox as single agents and in combination. DiOHF reduced Dox dose required to obtain identical decrease in cellular reductive metabolism, pointing to DiOHF potential in combination with Dox. Moreover, in the U2OS cell line, DiOHF–Dox combinations showed synergistic effects in the decrease in cellular reductive metabolism, pointing to enhanced anti-OS activity. In two previous studies, in the same cell line, synergistic interactions were reported between Dox (1–2.5 µmol/L, 48 h) and the flavonoids epigallocatechin-3-gallate and luteolin [25,36].

In this work, DiOHF decreased cyclin B2 expression in MG-63 cells, associated with accumulation in G2 phase. Cyclin B2 is essential for G2-phase exit and for the onset of mitosis [37] and in this study cyclin B2 expression was positively associated with cell percentage in G1 phase and negatively associated with cell percentage in G2 phase in both cell lines. Cdk1 is the only essential cyclin-dependent kinase and drives the cell cycle, promoting S/G2 and G2/M phase transitions [38]. Considering this, DiOHF decreased Cdk1 expression in the MG-63 cell line, and this was associated with increase in S-phase population, which is in line with Cdk1 role in the cell cycle.

Some studies have described the anti-cancer activity of flavonoids with structural similarities with DiOHF in several OS cells, including the flavonoid quercetin, with additional hydroxylation at the C-5 and C-7 positions in the B-ring. Lan et al. reported that incubation with quercetin (100 µmol/L, 24 h) did not lead to significant apoptosis or cell cycle arrest in this cell line [22]. In the study of Catanzaro et al. [39], the cytotoxic effects and cell cycle modulation of quercetin was tested in OS human cell line U2OS, and in its cisplatin-resistant counterpart, U2OSPt cells. It was found that in the U2OS cell line, the G0/G1 phase was increased by quercetin (10 µM and 50 µM), compared with the control, and the other phases reduced. In U2OSPt cells, the G0/G1 phase was lower than the M phase. Cyclin D1 expression was significantly reduced following the treatment with quercetin (50 µM) in U2OSPt cells, but not in U2OS cells. In both cell lines, cyclin B1 levels were not affected when treated with quercetin (50 µM. Other work [21] suggested that quercetin reduced metastatic OS cell invasion, adhesion, proliferation, and migration in U2OS and Saos-2 cells, by inhibiting parathyroid hormone receptor 1 (PTHR1). In the highly metastasizing human OS cell line, 143B, quercetin induced growth inhibition, G2/M phase arrest, and apoptosis [40]. In what concerns the effects of the 5,7-dihydroxyflavonol, known as galangin, on OS cells, it was observed that this compound decreased proliferation of MG-63 cells, and significantly enhanced apoptosis in this cellular line. Additionally, phosphoinositide-3 kinase (PI3K) and Aktp-Thr308 expression levels decreased, as well as the levels of cyclin D1 and matrix metalloproteinase (MMP) 2/9, in MG63 cells treated with galangin. In contrast, it was found that the expression levels of p27 and caspases-3 and -8 were downregulated [41]. Regarding apigenin, a trihydroxyflavone, it was described that the treatment of MG-63 cells (p-53 mutant cell line) with this flavonoid resulted in inhibition of growth and G2/M phase arrest, associated with induced p21/WAT expression [42]. Flavonoid ampelopsin has, like DiOHF, -OH groups at C-3, C-3′ and at C-4′, but also additional -OH substituents at C-5, C-5′, and at C-7. After exposure of MG63 cells to this flavonol, it was found a reduced viability in a time- and dose-dependent manner (20–100 µmol/L), as well as an increased apoptotic index. Ampelopsin blocked MG-63 cells at G0/G1 phase of the cell cycle, with increased p21CIP1 expression but decreased cyclin A and Cdk2 expression levels.

Concerning cell cycle effects in U2OS cells, the flavonoids icarisid II (20 µmol/L, 48 h) and pelargonidin (30 µmol/L, 48 h) arrested this cell line in G2/M phase [43,44]. In the case of incubation with pelargonidin, cytotoxicity was associated with G2/M phase arrest and autophagy [44]. In the case of icarisid II, incubation with this compound was associated with decreased expression of phosphorylated M-phase inducer phosphatase 3 (p-Cdc25c), responsible for dephosphorylation of cyclin B-bound Cdk1 and consequent entry into mitosis [43]. In our work, a non-significant decrease in cyclin B2 expression was observed in the same cell line, and considering the results observed for icarisid II, it cannot be excluded that the flavonoid DiOHF also decreased p-Cdc25c expression. In OS, a majority of differentially expressed genes were described as being involved in cell cycle regulation, including Cdk1 and cyclin B2, which were found upregulated in this tumor [45,46]. Multiple cellular alterations are responsible for differential Cdk1 and/or cyclin B expression. Huang et al. [47] reported that in OS cells, including the U2OS cell line, miR-199a-3p was downregulated, which in turn, elevated the expression of four miR-199a-3p targets which are crucial effectors of G2/M transition: Cdk1, cyclin B, aurora kinase A (AURKA) and Serine/threonine-protein kinase Nek2. Another epigenetic alteration resides in the long non-coding RNA lncRNA GAS5, which is downregulated in different OS cell lines, including the MG-63 cell line [48]. As reported by the authors, lncRNA GAS5 is a natural growth-suppressor miRNA sponge that decreases miR-203a levels. In MG-63 cells, miR-203a targeted the tissue inhibitor of metalloproteinases 2 (TIMP2) expression, and low TIMP2 levels resulted in increased activation of PI3K/AKT/GSK3-β and downstream activation of Cdk1, Cdk4, cyclin B and cyclin D expression [48]. Importantly, upregulation of lncRNA GAS5 induced by flavonoids, with consequent inhibition of cancer proliferation, has previously been reported [49]. Similarly, other proliferation pathways were associated with increased expression of cell cycle regulators in OS. In MG-63 cells, overexpression of fibroblast growth factor receptor 1 (FGFR1), a tyrosine-kinase receptor, increased Cdk1 levels, while PDCD5, which inhibited Ras/Raf/MEK/ERK pathway, decreased Cdk1 and cyclin B expression [50,51]. Interestingly, the DNA-binding cell division cycle 5-like protein (CDC5L) is overexpressed in MG-63 and U2OS cells, and its silencing induces G2/M cell cycle arrest associated with decreased Cdk1 and cyclin B expression [52,53]. In this study, incubation with DiOHF resulted in delayed nuclear division rates in MG-63 and U2OS cells. Previously, the flavonoid fisetin, with additional hydroxylation at C-7 in the B ring compared to DiOHF, was reported to induce a switch from cytoprotective autophagy to autophagic cell death, presumably through the induction of mitotic catastrophe in various cancer cell lines [54,55]. In the reported cell lines (A549, DU145, HeLa, MCF-10A, MCF-7, PC3), fisetin significantly perturbed spindle checkpoint signaling, presumably in a proteasome-dependent process and through the direct inhibition of aurora kinase activity [46]. It is noteworthy that the perturbation of nuclear division and the increased percentage of cell in G2/M phase found in this study do not exclude the possibility of other mechanisms of action, including inhibition of aurora kinase activity as reported for fisetin in other cell lines.

## 4. Materials and Methods

### 4.1. Cell Culture Media and Reagents

Dulbecco’s modified Eagle’s medium (DMEM), fetal bovine serum (FBS), L-glutamine, trypsin-EDTA (0.25% Trypsin, 1 mM EDTA) and penicillin-streptomycin were obtained from Thermo Fisher Scientific (Carlsbad, CA, USA). MTT, dimethyl sulfoxide (DMSO), methanol, Dox hydrochloride, sodium dodecyl sulphate (SDS), propidium iodide (PI), phosphate buffered saline (PBS), and ribonuclease were purchased from Sigma-Aldrich (St. Louis, MO, USA). DiOHF and CV were purchased from Merck Millipore (Billerica, MA, USA).

### 4.2. Cell Culture and Exposure Conditions

Human OS cell lines MG-63 and U2OS were authenticated by Short-Tandem Repeat (STR) profiling and purchased from American Type Culture Collection (ATCC, Rockville, MD, USA) in December 2009. Cells were immediately thawed, and a cell bank was cryopreserved from 3 subsequent passages. Cells were thawed from the initial cell bank and passaged for fewer than 4 months after resuscitation (passage numbers 27 to 54). The cell lines were maintained and subcultured in complete DMEM with 10% FBS, 100 U/mL penicillin, 100 µg/mL streptomycin, and 2 mM L-glutamine. Both cellular models were then incubated at 37 °C in a 5% CO_2_ humidified atmosphere. Subconfluent cells were harvested with trypsin-EDTA and then subcultured. To perform the exposure experiments, stock solutions of DiOHF (100 mM) were prepared in DMSO. For all conditions, the final concentration of DMSO used was 0.1%, including DMSO solvent control.

### 4.3. Cytotoxicity Assays

For MTT and CV assays, cells were plated at a density of 1 × 10^4^ cells/well in 96-well plates. After overnight adhesion, cells were treated with DiOHF (5, 10, 20, 40 and 100 μmol/L) or Dox (0.1, 0.2, 0.4, 0.8 and 1.6 μmol/L) for 48 h. The MTT assay, adapted to 96-well plates, was performed as previously [56]. Briefly, 2 h before the end of exposure, MTT reagent (final concentration 0.5 mg/mL) was added to each well and cells were further incubated at 37 °C for the remaining exposure time. After this, the medium was discarded, and the intracellular MTT formazan crystals were dissolved with addition of 100 μL DMSO per well. The absorbance values were measured at 570 nm using a multi-mode microplate reader (Synergy HT, Bio-Tek Instruments, Winooski, VT, USA). The CV assay allows to measure cell growth based on the determination of CV bound to DNA and proteins. Upon exposure to DiOHF, the medium was replaced with absolute ethanol (10 min fixation). Then, fixed cells were stained by addition of an equal volume of 0.1% CV solution followed by 30 min incubation. Plates were carefully washed with distilled water, dried and 1% SDS was added to each well followed by shaking for 1 h. Absorbance was measured at 590 nm in Synergy HT Multi-mode Microplate Reader (Bio-Tek Instruments, Winooski, VT, USA).

### 4.4. Drug Combination Analysis

Cells were seeded at a density of 1 × 10^4^ cells/well in 96-well plates. After overnight adhesion, cells were incubated with DiOHF (39.5, 59.3, 133 and 200 μmol/L), Dox (0.222, 0.333, 0.75 and 1.125 μmol/L) or their respective increasing DiOHF:Dox combinations: 39.5:0.222 µmol/L; 59.3:0.333 µmol/L; 133:0.75 µmol/L; 200:1.125 µmol/L. After MTT assay, the fractional inhibition (Fa) of MTT reduction at each dose was used to compute the dose reduction index (DRI) and combination index (CI) values, according to the Chou-Talalay method and using Compusyn software [57].

### 4.5. Cell Apoptosis and Necrosis

Quantitative assessment of apoptosis and necrosis was performed by flow cytometry using fluorescein isothiocyanate (FITC)-Annexin V and PI staining. Briefly, 1 × 10^5^ MG-63 cells/well or 2 × 10^5^ U2OS cells/well were seeded in 6-well plates containing complete DMEM and left to adhere overnight. After exposure to DiOHF below IC_50_ concentrations, cells were briefly washed with PBS and harvested by trypsinization. From this step, the FITC Annexin V Apoptosis Detection Kit I (BD Pharmingen, San Diego, CA, USA) was used according to the manufacturer’s instructions. Samples were excited with a 488 nm laser, at least 10,000 gated events were analyzed in a BD Accuri C6 instrument (BD Biosciences, San Jose, CA, USA) in FL1 and FL3 channels. Percentages were calculated from the number of cells in each quadrant divided by the total number of cells, using the BD Accuri C6 software.

### 4.6. Cell Cycle Analysis

Cells (1 × 10^5^ MG-63 cells/well or 2 × 10^5^ U2OS cells/well) were seeded in 6-well plates containing complete DMEM and left to adhere overnight. After exposure to DiOHF below IC_50_ concentrations (Table 1), cells were washed with PBS, harvested after trypsinization, centrifuged (700× *g*, 5 min), and resuspended in PBS. Cell suspensions were centrifuged (300× *g*, 5 min), the pellet resuspended in 85% ethanol and stored at −20 °C until analysis. On the day of analysis, ethanol solution was removed after centrifugation (800× *g*, 5 min), and fixed cells were resuspended in PBS. Cell suspensions were filtered through a 35-µm pore nylon mesh, and RNase and PI, both at 50 µg/mL final concentration, were added. Stained samples were analyzed in a flow cytometer (Attune Acoustic Focusing Flow Cytometer, Thermo Fisher Scientific, Waltham, MA, USA). This equipment was provided with a violet laser tuned at 50 mW and operating at 405 nm and a blue laser tuned at 20 mW operating at 488 nm. FlowJo (Tree Star Inc., Ashland, OR, USA) was used to analyze cell cycle distribution.

### 4.7. Cytokinesis-Block Micronucleus (CBMN) Cytome Assay

Cells, 1 × 10^5^ MG-63 cells/well or 2 × 10^5^ U2OS cells/well, were seeded on cover glasses in 6-well plates containing complete DMEM and left to adhere overnight. After exposure to DiOHF, the exposure medium was replaced with DMEM containing 3 µg/mL cytochalasin B and cells were incubated for 40 h. The CBMN assay was a modification of Fenech’s protocol [58], conducted according to the OECD guideline 487 (In Vitro Mammalian Cell Micronucleus Test, 2016). After exposure, cells on cover glasses were washed with PBS, methanol-fixed at −20 °C for 10 min, dried, and stained with acridine orange. The samples were observed under an Eclipse 80i fluorescence microscope (Nikon, Tokyo, Japan), equipped with a B-2A filter. Cytokinesis-block proliferation index (CBPI) values were calculated according to the OECD guideline 487 (2016) (https://www.oecd.org/chemicalsafety/test-no-487-in-vitro-mammalian-cell-micronucleus-test-9789264264861-en.htm (accessed on 14 January 2020)) for cytochalasin B treated cells.

### 4.8. RNA Extraction, Reverse Transcription and qPCR

Oligonucleotide primers (Table S1) were designed using the Primer3 platform [59] and primer specificity was confirmed with the UCSC In-Silico PCR tool (http://genome.ucsc.edu/cgi-bin/hgPcr?command=start(accessed on 2 February 2020). Cells (1 × 10^5^ MG-63 cells/well or 2 × 10^5^ U2OS cells/well) were seeded in 6-well plates containing complete DMEM and left to adhere overnight. After DiOHF exposure and washing with PBS, cells were lysed in NZYol (NZYtech, Lisbon, Portugal) and RNA was extracted according to the manufacturer’s instructions. Turbo DNA-free kit (Ambion, Austin, TX, USA) was used to eliminate possible genomic DNA contamination in RNA samples. For cDNA synthesis, 1 μg total RNA was reverse-transcribed using the NZY First-Strand cDNA Synthesis Kit (NZYTech, Lisbon, Portugal). Before qPCR, the cDNA samples were prediluted in ultrapure water (1:5). The final individual qPCR reactions contained NZYSpeedy qPCR Green Master Mix (2×) (NZYTech, Lisbon, Portugal), gene-specific primer (150 nM each) and prediluted cDNA [1:4 (*v*/*v*)]. The program of qPCR comprised denaturation for 1 min at 95 °C, and 60 cycles of amplification that included denaturation for 5 s at 94 °C, and annealing with extension for 30 s at 60 °C. After qPCR, a melting temperature program was performed. At least three qPCR technical replicates were performed per sample from each of three independent experiments. Quantification cycles were extracted from CFX96 Real-Time PCR Detection System (Biorad, Hercules, CA, USA). Gene expression of exposed cells was calculated relative to control cells and normalized with the GAPDH reference gene, following the 2^−ΔΔCT^ method [60].

### 4.9. Statistical Analysis

All data were derived from at least three independent experiments (*n* ≥ 3). The inhibitory concentrations (ICs) and dose-response curves were determined with CompuSyn software [57]. Results are mean ± standard deviation (SD). GraphPad Prism 6 software (San Diego, CA, USA) and SigmaPlot software for windows (version 11.0, Systat Software Inc, San Jose, CA, USA) were used for graphical representation and to compare control vs. experimental groups by one-way analysis of variance (ANOVA) with post-hoc Holm-Šidák test. For all comparisons, differences were considered statistically significant for significance levels of *p* < 0.05, *p* < 0.01, and *p* < 0.001.

## 5. Conclusions

The limitations currently observed in OS treatment due to common chemoresistance may be prevented by applying combined therapy. In the present study, OS cellular models, U2Os and MG-63, were treated with DiOHF, Dox and respective combination. From the obtained results, it was found that DiOHF was able to reduce OS cell growth and viability in a concentration-dependent manner. The combination of this flavonoid with Dox allowed a decrease in Dox dose in both studied cellular lines, with observed synergistic interactions in U2Os cells. In addition to the cytostatic effects in U2OS and MG-63 cells, treatment with DiOHF modified cell cycle dynamics and provided differential cyclin and cyclin-dependent kinase expression. In conclusion, the antiproliferative effects observed herein indicate a positive role of DiOHF in therapeutic regimens in OS. In combination with Dox anthracycline, the cardioprotective agent DiOHF showed beneficial effects in the dose reduction of this drug, with potential consequences in the alleviation of Dox cardiotoxicity and prevention of chemoresistance in OS patients.

## Figures and Tables

**Table 1:** IC_50_ values (µmol/L ± SD) of DiOHF and Dox. Cells were exposed to DiOHF or Dox for 48 h and IC_50_ values were determined following MTT and CV assays.

Cell Line	Mean IC_50_ (μmol/L) ± SD ^1^			
	MTT ^2^ Assay		CV ^3^ Assay	
	DiOHF ^4^	Dox ^5^	DiOHF	Dox
MG-63	103 ± 41	0.750 ± 0.101	50.3 ± 1.6	0.475 ± 0.136
U2OS	93.3 ± 19.8	0.434 ± 0.078	72.1 ± 12.3	0.454 ± 0.046

^1^ SD—standard deviation; ^2^ MTT—3-(4,5-Dimethyl-2-thiazolyl)-2,5-diphenyl-2*H*-tetrazolium bromide; ^3^ CV—crystal violet; ^4^ DiOHF—3′, 4′-dihydroxyflavonol; ^5^ Dox—doxorubicin.

## Data Availability

Data is contained within the article and Supplementary Material.

## Supplementary Materials

The following are available online at https://www.mdpi.com/article/10.3390/ph14070640/s1, Figure S1: Effects of DiOHF on apoptosis, Table S1: Oligonucleotide primer sequences used for quantitative PCR.
