# Supplementary material for: 3′,4′-Dihydroxyflavonol Modulates the Cell Cycle in Cancer Cells: Implication as a Potential Combination Drug in Osteosarcoma
Source: Pharmaceuticals (Basel). 2021 Jul 3;14(7):640. doi: 10.3390/ph14070640 (PMC8308859; doi:10.3390/ph14070640)
Supplement: Supplementary file 1 [file pharmaceuticals-14-00640-s001.zip › pharmaceuticals-1274304-supplementary.pdf]

Supporting Information

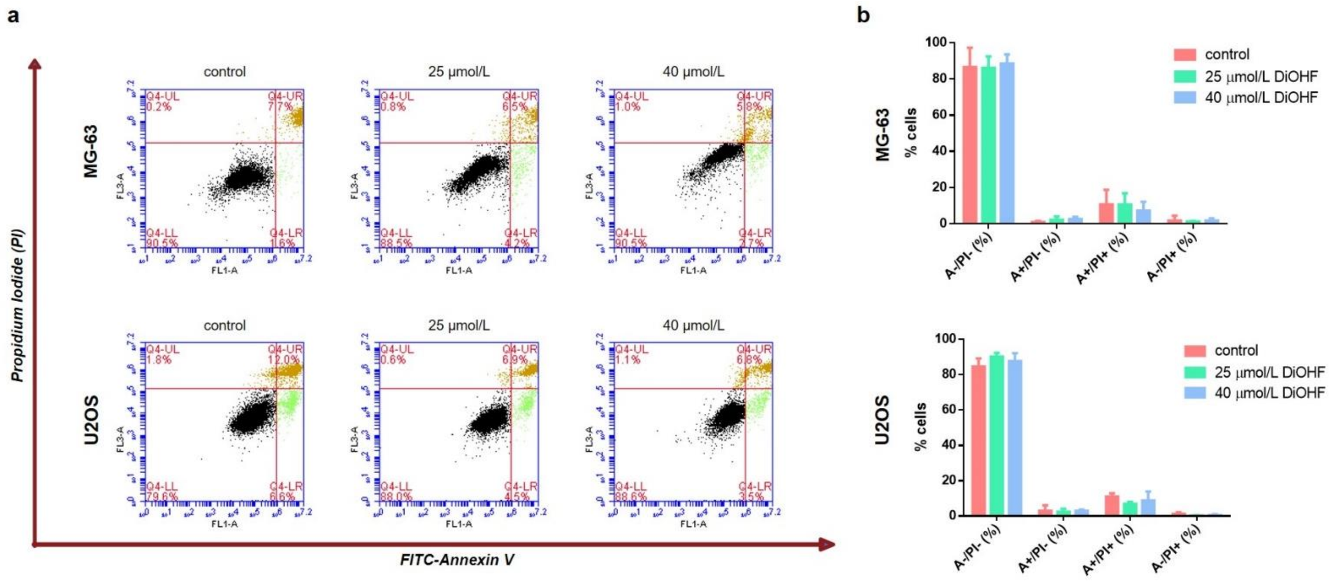

Figure S1. Effects of DiOHF on apoptosis. After 48h incubation with DiOHF, MG-63 and U2OS cells were harvested, incubated with FITC-annexin V and propidium iodide, and analysed by flow cytometry. a) representative dot plot diagrams of osteosarcoma cells labelled with FITC-annexin V (FL1) and propidium iodide (FL3); b) percentage of cells identified as FITC-annexin-V / PI: - / - (viable), + / - (early apoptotic), + / + (late apoptotic + necrotic) and - / + (necrotic). The data are mean ± SD (N=3). No significant differences were detected (one-way ANOVA).

Table S1. Oligonucleotide primer sequences used for quantitative PCR.

Gene	Oligonucleotide primer sequence (5'-3')
CCNB2 (cyclin B2)	F:GTCGACCCTTGCCACTACAC;<br>R:CCTAGAACCTTCTGAGACAAGCA
CCNE1 (cyclin E1)	F:CAGCCTTGGGACAATAATGC;<br>R:GAGGCTTGCACGTTGAGTTT
CDK1 (cyclin-dependent kinase 1)	F:GGGTAGACACAAAACTACAGGTCAA;<br>R:GGAATCCTGCATAAGCACATC
CDK2 (cyclin-dependent kinase 2)	F:ACCTCCCCTGGATGAAGATG;<br>R:AGATGGGGTACTGGCTTGGT
GADPH (glyceraldehyde 3-phosphate dehydrogenase)	F:ACACCCACTCCTCCACCTTT;<br>R:TACTCCTTGGAGGCCATGTG
